# Correction: Chen et al. Multivariate Framework of Metabolism in Advanced Prostate Cancer Using Whole Abdominal and Pelvic Hyperpolarized 13C MRI—A Correlative Study with Clinical Outcomes. *Cancers* 2025, *17*, 2211

**DOI:** 10.3390/cancers17152511

**Published:** 2025-07-30

**Authors:** Hsin-Yu Chen, Ivan de Kouchkovsky, Robert A. Bok, Michael A. Ohliger, Zhen J. Wang, Daniel Gebrezgiabhier, Tanner Nickles, Lucas Carvajal, Jeremy W. Gordon, Peder E. Z. Larson, John Kurhanewicz, Rahul Aggarwal, Daniel B. Vigneron

**Affiliations:** 1 Department of Radiology and Biomedical Imaging, University of California, San Francisco, CA 94158, USA; robert.bok@ucsf.edu (R.A.B.); michael.ohliger@ucsf.edu (M.A.O.); zhen.wang@ucsf.edu (Z.J.W.); daniel.gebrezgiabhier@ucsf.edu (D.G.); tanner.nickles@ucsf.edu (T.N.); lucas.carvajal@ucsf.edu (L.C.); jeremy.gordon@ucsf.edu (J.W.G.); peder.larson@ucsf.edu (P.E.Z.L.); john.kurhanewicz@ucsf.edu (J.K.); dan.vigneron@ucsf.edu (D.B.V.); 2 Helen Diller Family Comprehensive Cancer Center, University of California, San Francisco, CA 94158, USA; ivan.dekouchkovsky@ucsf.edu (I.d.K.); rahul.aggarwal@ucsf.edu (R.A.)

## Error in Figure

In the original publication [1], there was a mistake in Figures 2 and 5 as published. Figures with an old, obsolete k_PL_ unit (s^−1^) were erroneously uploaded during editing and proofreading. These figures are now corrected and have been replaced with k_PL_ unit ks^−1^, which is the preferable style. The corrected Figure 2 and Figure 5 appear below. The authors state that the scientific conclusions are unaffected. This correction was approved by the Academic Editor. The original publication has also been updated.

## Figures and Tables

**Figure 2:**
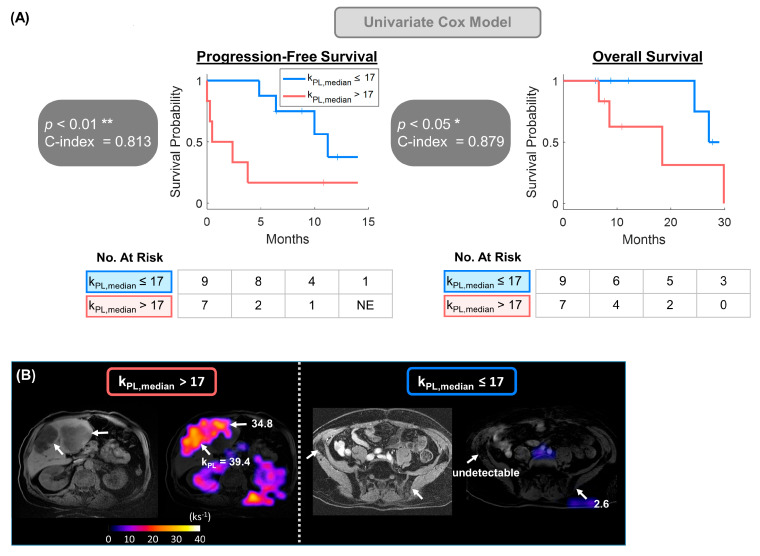
HP multiparametric features of metabolism (MFM) k_PL,median_ was selected and applied to univariate Cox proportional hazards model. (**A**) Kaplan–Meier analysis revealed substantially longer PFS and OS in patients with k_PL,median_ ≤ 17 ks^−1^ (median PFS: 11.2 vs. 0.5 months; median OS: NR vs. 18.4 months). (**B**) Example cases illustrating metastatic prostate cancer patients with k_PL,median_ higher and lower than the dichotomized cutoff value of 17. The patient on the left had high-volume liver metastases with k_PL,median_ = 24.8, whereas on the right had low or undetectable k_PL,median_ across the osseous pelvic metastases. Images shown were k_PL_ heatmaps overlaid on ^1^H T_1_-FSPGR references. * *p* < 0.05, ** *p* < 0.01.

**Figure 5:**
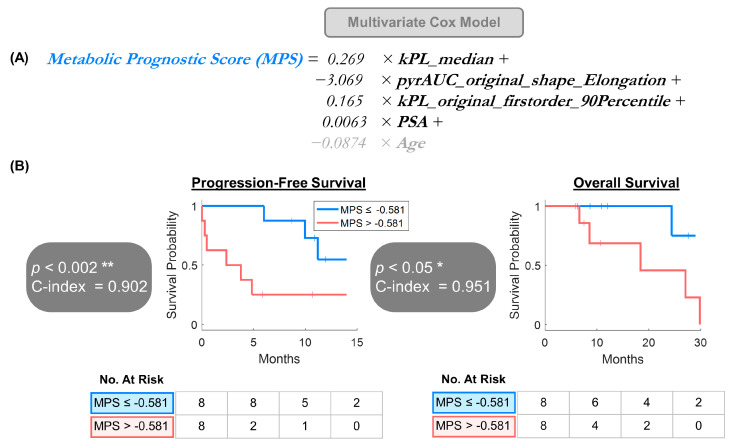
Hypothesis-generating multivariate survival analyses using (**A**) metabolic prognostic score (MPS) derived from multivariate Cox proportional hazards model, adjusted for patient age. (**B**) MPS was significantly associated with both PFS (*p* < 0.002) and OS (*p* < 0.05), with longer median PFS (NR vs. 2.4 months) and OS (NR vs. 18.4 months) for lower-MPS vs. higher-MPS patients, dichotomized by the cohort median MPS = −0.581. * *p* < 0.05, ** *p* < 0.01.
