# Supplementary material for: Multivariate Framework of Metabolism in Advanced Prostate Cancer Using Whole Abdominal and Pelvic Hyperpolarized 13C MRI—A Correlative Study with Clinical Outcomes
Source: Cancers (Basel). 2025 Jul 1;17(13):2211. doi: 10.3390/cancers17132211 (PMC12248915; doi:10.3390/cancers17132211)
Supplement: Supplementary file 1 [file cancers-17-02211-s001.zip › cancers-3593304-supplementary.pdf]

Multivariate Framework of Metabolism in Advanced Prostate Cancer using Whole Abdominal and Pelvic Hyperpolarized ^{13}C MRI – A Correlative Study with Clinical Outcomes

	k_{PL}median	k_{PL}max	k_{PL}kurtosis	TMV	PSA	LDH	ALP	PFS	OS
k_{PL}median	1.000	0.867	0.596	0.376	0.083	0.082	0.540	-0.317	-0.455
k_{PL}max		1.000	0.741	0.618	0.123	-0.090	0.254	-0.484	-0.542
k_{PL}kurtosis			1.000	0.585	0.022	-0.052	0.214	-0.745	-0.623
TMV				1.000	0.548	-0.016	0.023	-0.413	-0.360
PSA					1.000	-0.241	-0.025	-0.569	-0.541
LDH						1.000	0.049	0.174	0.433
ALP							1.000	0.347	0.025
PFS								1.000	0.776
OS									1.000

Supplemental Figure S1 Numerical representation of the Pearson correlation matrix in Figure 3. Pink-filled entries show statistically significant ($p<0.05$) correlations.

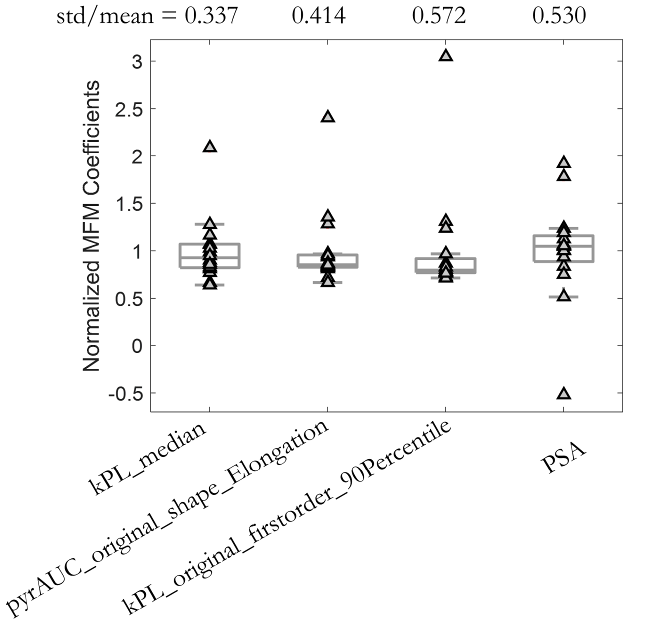

Supplemental Figure S2 Sensitivity analysis of multivariate risk variables (i.e. MFM) in a cross validation manner. By withholding one patient dataset at a time and evaluating the variability of model coefficients, the calculated normalized standard deviation of these MFMs ranged from 0.33 to 0.57.
